# Colo-Fallopian Fistula: A Rare Complication of Sigmoid Colon Diverticulitis

**DOI:** 10.7759/cureus.43331

**Published:** 2023-08-11

**Authors:** Daemar H Jones, Simmone M Spielmann, Sirin Falconi, Izi Obokhare

**Affiliations:** 1 General Surgery, Texas Tech University Health Sciences Center, Lubbock, USA

**Keywords:** gastrointestinal disease, lower gi or colorectal surgery, colo-fallopian fistula, colorectal surgery, sigmoid diverticulitis

## Abstract

Diverticulitis is a common colorectal disease present in Western countries that develops as infected protrusions (diverticula) along weak points in the colon due to increased intraluminal pressure. Most patients with diverticular disease can be asymptomatic; however, several complications can arise from the development of diverticulitis. Here, we discuss the diagnosis and management of a patient presenting with recurrent *Escherichia coli* (*E. coli*) vaginal infections due to sigmoid colon diverticulitis resulting in a colo-fallopian fistula that was unremarkable on diagnostic imaging. The patient was managed with minimally invasive surgery.

A 65-year-old female with a medical history of hyperlipidemia and recurrent diverticulitis presented with over a year history of recurrent *E. coli* vaginal infections. She underwent a robotic anterior resection with extracorporeal colorectal anastomosis via a Pfannenstiel incision. Less than 48 hours following the surgery, she was discharged without complications and has remained symptom-free nine months postoperatively. Significant improvement was noted following the procedure. The patient was able to advance her diet and was discharged the next day. The patient was seen postoperatively, with no evidence of any recurrent *E. coli* vaginal infections. The case highlights the diagnosis and management of a rare case of colo-fallopian fistula in a situation where the patient had recurrent vaginal infections. It is quite difficult to identify the fistula radiologically. This patient was managed with a minimally invasive surgical technique that proved to be safe and beneficial to the outcome of this patient.

## Introduction

Diverticula are characterized by the formation of outpouchings or herniations throughout the large intestine and are more prevalent in older patients in the Western world [1,2]. The pronounced diverticula are often found incidentally during colonoscopies as asymptomatic diverticulosis. Several factors can contribute to diverticula formation, including but not limited to colonic dysmotility, fiber intake, genetics, vitamin D, and level of physical activity [3]. Once diverticula become inflamed, they will lead to diverticulitis. Fistula secondary to diverticulitis is a possible chronic complication that starts with nonspecific symptoms [2,4]. Colo-fallopian fistulas, although extremely rare, should be suspected in female patients with recurring vaginal discharge and urinary tract infections (UTIs). We present a case of the development of a colo-fallopian fistula in an asymptomatic patient with recurrent vaginal infections that was managed surgically and a subsequent review of the literature.

## Case presentation

**Figure 1:**
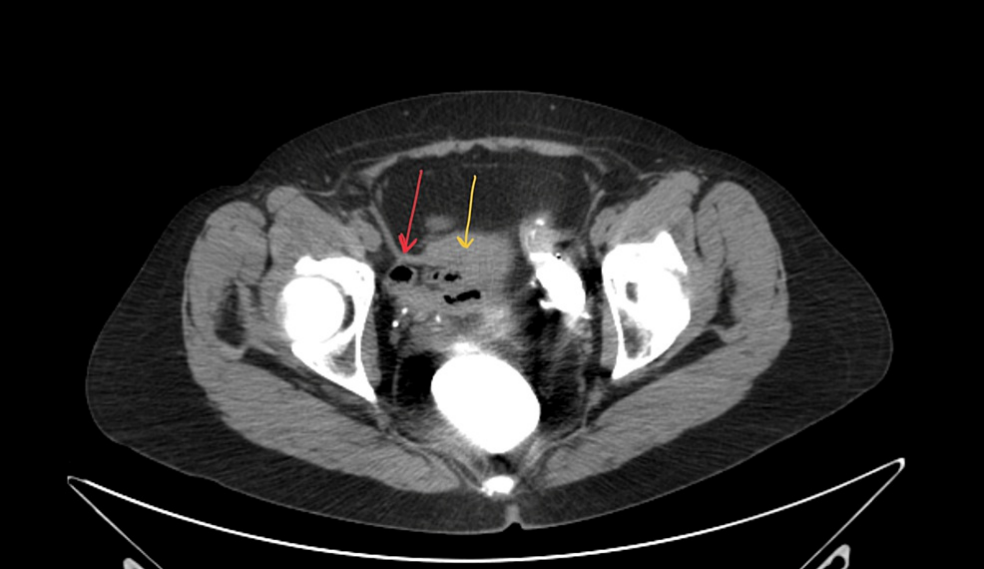
Axial CT scan with contrast of the pelvis shows the uterus (yellow arrow) and right fallopian tube (red arrow), which failed to show the fistula. CT: computed tomography

**Figure 2:**
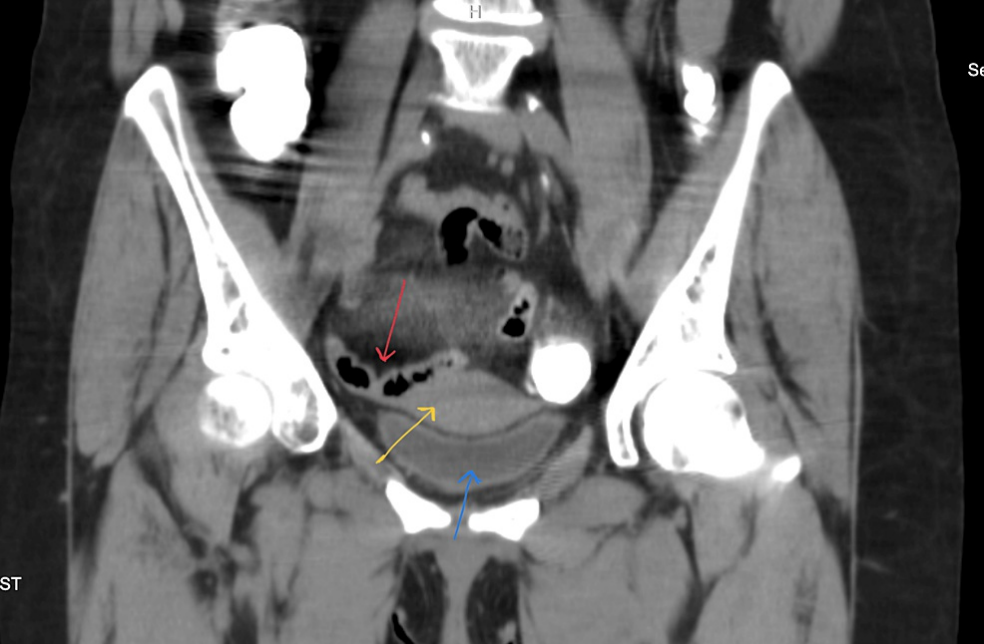
Coronal CT scan with contrast of the pelvis shows the bladder (blue arrow), uterus (yellow arrow), and air in the small bowel loop (red arrow) with inflammatory changes in fat stranding in the middle (haziness). CT: computed tomography

A 65-year-old female with a past medical history of diverticulosis and hyperlipidemia presented to the clinic with recurrent vaginal discharge for over a year. During this course, she was managed with antibiotic treatment by her primary care physician. She continued to have recurrent malodorous discharge despite antibiotic treatment. Urine analysis was negative, and she was referred to a gynecologist and colorectal surgeon. She denied any fever or abdominal pain. A computed tomography (CT) scan with rectal contrast and colonoscopy was unremarkable and failed to show any suspecting fistula (Figures 1, 2). However, due to her history of recurrent diverticulitis and repeated positive cultures of *E. coli* from her vagina, coupled with a family history of complicated diverticulitis, she was scheduled for a diagnostic laparoscopy with cystoscopy and possible sigmoidectomy.

Cystoscopy and bilateral ureteral stent placement were performed after the perianal area was prepped. On brief examination of the bladder, there were no lesions or fistulas noted. A diagnostic laparoscopy was performed, which showed a normal-appearing gallbladder and liver. No evidence of injury upon entry was noted. The sigmoid colon was noted in the pelvic area with adhesions to the uterus and to the anterior abdominal wall. There were some dense adhesions to the right fallopian tubes suggestive of the location of the colo-fallopian fistula. Laparoscopically, the sigmoid mobilization was performed in the medial to lateral fashion with protection of both ureters. High ligation of the inferior mesenteric artery was performed to further dissect the sigmoid colon, rectosigmoid junction, and mid-rectal area. Adequate vascularity to the descending colon was ensured using the indocyanine green (ICG) and Firefly technology mesenteric angiography. The sigmoid colon and mid-rectum were brought out through a Pfannenstiel incision for the sigmoidectomy. The descending colon, sigmoid colon, and mid-rectum were brought out through the incision. An end-to-end anastomosis was created between the distal rectum and descending colon. A leak test was performed in the pelvis, which showed no evidence of an anastomotic leak. The anastomotic donuts were intact. The anastomotic donuts, sigmoid colon, and rectum were sent for pathological examination. Microscopic evaluation confirmed diverticulosis and diverticulitis with no evidence of malignancy or perforation. Postoperatively, the patient was able to advance to a soft diet the next day. She later tolerated a solid diet and was ambulating well. She followed up in two weeks with no evidence of recurrent *E. coli* vaginal infections.

## Discussion

Diverticular disease is becoming more common in Western countries, especially with increased age. The formation of multiple diverticula can lead to incidental findings of asymptomatic diverticulosis. Diverticulitis is a complication that occurs when a fecalith obstructs the diverticula, leading to irritation and inflammation of the mucosa [5]. The majority of diverticulitis complications are associated with abscess formation, with colonic perforation or obstruction occurring less commonly [3,5]. Colonic fistulas are among these other complications that can present initially in a well-appearing patient. Although not fully understood, it is believed that adhesions between two structures may occur during an episode of acute diverticulitis [3]. Fistula formation is also thought to occur as a result of abscess formation. The abscess will perforate the colon, allowing fistulas to form due to the weakening of adjacent structures.

Sigmoid fistulas are difficult to diagnose. Clinical manifestations can vary, and symptoms are nonspecific. Colo-fallopian fistulas can present with recurrent vaginal discharge as a significant finding. Patients can also have multiple positive tests for bacterial vaginal infections. Guidelines vary from both a gynecologic and surgical standpoint [6]. Gynecologists may use transvaginal ultrasound or hysteroscopy to visualize a fistula in the uterus. Triple-contrast abdominal CT is a recommended option for diverticulitis, but the findings for fistula formation may not be clear. This is a possible disadvantage to recognizing and treating sigmoid fistulas appropriately. Hysterosalpingography and colonoscopy are notable in helping diagnose colo-fallopian fistulas in particular [6]. As fistula connections can form in a variety of locations, the use of more than one imaging modality may be necessary before ruling out the presence of a fistula.

Patients that present with acute complications of diverticulitis can be treated conservatively. However, since diverticula are still present, patients remain prone to recurrences of diverticulitis that may progress to chronic complications [3-7]. The surgical approach is a solution known to resolve a patient’s symptoms [7]. Unstable patients usually require an urgent Hartmann procedure that removes the sigmoid colon with end colostomy because of the overwhelming infection causing shock [3]. Alternatively, patients can be treated with an elective operation involving a primary anastomosis or end-to-end anastomosis [3,7,8]. This is an option for hemodynamically stable patients who were treated conservatively without improving symptoms, which is often preferred over the Hartmann procedure [8]. Fistulas should remain on the differential in patients with long-term diverticulitis. Patients should be aware of elective surgical options, along with conservative treatment.

Patients that are treated surgically are known to have a better improvement of their symptoms and quality of life compared to those treated conservatively. Some circumstances have reported refusal of surgical treatment or delayed treatment, which resulted in the patient’s untimely death [6,9]. There are also reported cases of colo-fallopian fistulas that were managed gynecologically. Our novelty is that our patient was able to be treated with minimally invasive techniques that improved not only her symptoms but also her quality of life.

## Conclusions

Colo-fallopian fistulas are a rare complication of long-standing diverticulosis and should be suspected in older female patients with recurring vaginal infections refractory to antibiotic treatment. The purpose of presenting this case is to keep colo-fallopian fistulas on the differential diagnosis in female patients with recurrent vaginal discharge, especially if there are a history or risk factors pointing to diverticular disease. Our case proves that recognizing and surgically treating fistula formation from complicated diverticulitis early on is lifesaving and beneficial to the patient’s health without any post-surgery complications.
